# ISIT: a frame-clustering implicit measure of situated experience

**DOI:** 10.3389/fpsyg.2026.1883850

**Published:** 2026-10-05

**Authors:** Aldo Gangemi, Chiara Lucifora

**Affiliations:** 1 Department of Philosophy and AlmaAI, University of Bologna, Bologna, Italy; 2 Institute of Cognitive Sciences and Technologies, National Research Council, Rome, Italy; 3 Department of Human Sciences, Link Campus University, Rome, Italy

**Keywords:** computational grounded theory, frame semantics, implicit knowledge extraction, knowledge graphs, situated cognition, verbal reports

## Abstract

**Introduction:**

The Implicit Situation Intensity Test (ISIT) is a method for converting participants’ verbal reports about an experience into a quantitative, per-participant *implicit measure* of how strongly cognitive, emotional, behavioral, and value-laden frames have been activated by that experience.

**Method:**

ISIT operates by clustering frames extracted from the verbal report’s *extended knowledge graph* (XKG)—the structured representation produced by the POLANYI++ neurosymbolic pipeline—into a Frame Ontology (FO) and scoring each frame’s salience for each participant on a normalized scale. The implicit measure produced by ISIT is designed to be combined with explicit measures from standardized questionnaires, implicit measures from physiological sensors, and participant profile information, in a single multivariate analysis of situated experience, producing *participant-level scores*, suitable for psychometric integration.

**Results:**

We illustrate the method through the Lucifora et al. study on virtual male embodiment in a catcalling scenario (*n* = 36), in which a 37-indicator Frame Ontology spanning four domains (emotional states, behavioral responses, violence perception, and semantic markers), whose frame clusters entered the study’s multivariate correlation structure alongside the standardized instruments and emotion ratings; safety-related frames showed differential correlation patterns with anger and sadness versus with fear that simpler instruments do not capture. To place the extraction itself on an auditable footing, every frame the pipeline produced was adjudicated by a tacit-aware judge (DEEPJUDGE) and human-verified: frame-level precision on a warrant criterion was high (0.95–0.96 across two foundation-model backends, with near-zero over-projection), and the two backends produced markedly more similar profiles for the same report than for different ones.

**Discussion:**

ISIT shares with grounded theory (GT) and computational grounded theory (CGT) the inductive commitment that situational structure should emerge from data, but it differs in three operational respects: it operates on structured extended knowledge graphs (XKGs) rather than raw text; it produces participant-level numerical scores rather than corpus-level qualitative themes; and it integrates by design with other measurement channels rather than standing as a free-standing qualitative analysis. ISIT fills the verbal-channel gap in the cognitive-science measurement ecosystem: it is the implicit-measure complement to autonomic, behavioral, and questionnaire-based instruments, not their replacement. Convergent and divergent validity against standard implicit measures, predictive validity against behavioral outcomes, and multi-modal integration validation are set out as a defined roadmap.

## 1 Introduction

Cognitive science measures situated experience primarily through three complementary channels. Standardized explicit instruments—Likert scales, categorical assignments, validated questionnaires—provide objective, reproducible numbers but operate under a closed-world assumption (Reiter, 1981). This assumption, originating in logic and artificial intelligence, posits that all relevant facts, variables, and categories are known and specified *a priori* by the experimenter. When a participant is asked to rate their emotional state on a 5-point Likert scale across six predefined emotions, the world of their experience is closed to any phenomena that fall outside those categories. This methodological choice reflects a positivist epistemology that treats psychological phenomena—motives, needs, emotions—as static entities to be objectively measured by a neutral observer, rather than as dynamic processes embedded in context. A closed world is a necessary simplification for many computational systems and psychological studies. Still it serves as a poor model for realistic, intelligent agents who navigate an open world, where the knowledge depends on more variables than those known in advance by the experimental setting.

In a closed world, phenomena outside the predefined categories are invisible, and the categories themselves carry the experimenter’s theoretical commitments without exposing them to the data. Physiological and behavioral sensors—galvanic skin response (GSK), heart-rate variability (HRV), eye-tracking, electroencephalography (EEG), functional magnetic resonance imaging (fMRI)—provide implicit indicators that bypass introspective access but are, by construction, indirect: they record bodily and neural correlates of experience, not its conceptual structure.

Verbal reports, the third channel, reveal an open world, and provide the conceptual content directly. However, the dominant verbal report analytical tradition—qualitative inductive coding—is laborious, low-throughput, and only partially reproducible across analysts (Glaser and Strauss, 2009; Charmaz, 2014).

In practice, only the first two channels are currently well-instrumented: standardized questionnaires and physiological sensors produce well-typed numerical outputs ready for inferential statistics. The third channel is not well-instrumented to be associated with the first two channels. For example, a study that elicits a 5-min retrospective report from each participant, produces a rich data stream whose conceptual content is opaque to standard inferential pipelines. The analyst is left to choose between (a) discarding the verbal report or treating it as a manipulation check; (b) coding it manually, accepting the throughput and reproducibility costs; or (c) reducing it to surface metrics—word count, sentiment polarity, topic distribution—that ignore the situational structure. None of these is satisfactory. The verbal channel is the channel through which the participant’s own conceptual framing of the experience is articulated; closing it to inferential analysis loses the data that situate the experience situated.

The Implicit Situation Intensity Test (ISIT) analyses the verbal channel without closing the open world. ISIT does not impose a fixed coding scheme; it does not require an *a priori* Frame Ontology. It takes as input the extended knowledge graph—produced from each participant’s verbal report by POLANYI++, a neurosymbolic AI pipeline (De Giorgis et al., 2025)—a graph whose nodes are entities including events, frames, persons, etc., and whose edges are typed semantic and causal relations, all annotated with the heuristic provenance that licensed each inference. ISIT then clusters the frames present in the graph into a Frame Ontology (either an externally supplied one or an ontology induced from the union of multiple participants’ graphs, in the multi-case configuration), and scores each frame’s salience for each participant on a normalized [−1, +1] scale. The output is a per-participant Situation Profile: a typed numerical vector indexed by frames in the Frame Ontology, suitable for direct integration with either explicit measures from questionnaires, or implicit measures from physiological sensors.

The relevant architectural relationship is as follows. POLANYI++ is the extraction backbone: it converts verbal text into a structured, audit-traceable knowledge graph, applying a battery of heuristics for tacit content (presupposition, implicature, multi-layer impact, semantic coercion, theory of mind, perspective, modality, causal reasoning, qualia composition) and an audit method (DARKSIDE) for via-negativa coherence checking. ISIT—formally specified as the method ISITPROFILE within the POLANYI++ method registry (De Giorgis et al., 2025)—operates on the output of POLANYI++ extraction, not on the raw text. The implicit measure ISIT produces is, in effect, a quantitative summary of the situational structure that POLANYI++ has surfaced. POLANYI++ also provides DEEPJUDGE, a tacit-aware adjudication method that verifies whether each extracted frame is warranted by the report; the present article uses it to place ISIT’s extraction on an audited accuracy footing (§5.1).

This study extends the original ISIT framework, first applied in earlier work on a male-embodiment catcalling study (Lucifora et al., 2025), along three dimensions. First, it makes explicit ISIT’s reliance on, and integration with, the POLANYI++ pipeline as the extraction backbone. Second, it positions ISIT against grounded theory and its computational variants—ISIT is not an automated GT; it is a frame-clustering implicit-measure method that shares only the inductive commitment with the GT family. Third, it specifies the integration recipe by which ISIT outputs combine with explicit and implicit measurements from other channels in a unified multivariate analysis of situated experience.

The study offers four contributions.

The first is the explicit integration of ISIT with the POLANYI++ knowledge graph extraction backbone. POLANYI++ uses heuristic and logical components to extract the implicit frames and situations evoked by a verbal report, jointly with a built-in auditing method to protect the extracted frames against the silent contradictions that bedevil verbal-report analysis. It also addresses the composition of those frames, ensuring that they are not bound to a single emotion or value theory but instead comprise multiple theoretical projections within an integrated extended knowledge graph (XKG; De Giorgis et al., 2025).

The second is the explicit distinction between ISIT and the grounded-theory family. ISIT shares with grounded theory the inductive commitment that situational structure should emerge from data, but it differs in three operational respects: it operates on structured XKGs rather than raw text; it produces participant-level numerical scores rather than corpus-level qualitative themes; and it integrates by design with other measurement channels rather than standing as a free-standing qualitative analysis.

The third is the specification of ISIT’s integration with other measurement channels. We document the integration recipe for combining ISIT outputs with explicit standardized instruments, with implicit physiological measures, and with participant profile information, in a single multivariate analysis. This integration is what makes ISIT useful in the cognitive-science measurement ecosystem rather than a free-standing tool.

The fourth is empirical anchoring. We demonstrate the architecture the published study by Lucifora et al. (2025) on virtual male embodiment in a catcalling scenario.

This article is a *Hypothesis and Theory* contribution. It proposes ISIT as a method, specifies its computational pipeline, situates it relative to grounded theory and to association-based implicit measures, and demonstrates its use through one published deployment. ISIT has been successfully used in at least one published peer-reviewed deployment (Lucifora et al., 2025), in which it produced interpretable frame profiles whose clusters correlated with the explicit measures at the cluster level and surfaced differential correlation patterns across Ekman emotion-intensity dimensions, while the strong convergent validity (r > 0.95) held among the standardized instruments themselves. We refer to this as *demonstrated feasibility under integration with established explicit measures*: the method ran end-to-end on a real cognitive-science corpus, produced typed numerical outputs, and the outputs entered into a multivariate analysis whose conclusions were independently reviewed and published. We do not treat this single deployment as evidence of convergent, divergent, or predictive validity of ISIT as a psychometric instrument. A formal psychometric validation program—with sample sizes adequate to the indicator count, with multiple-comparison correction, and with reliability estimation—is outlined as a roadmap in §5.1 and is the natural locus of subsequent work.

## 2 Background and related study

ISIT’s central unit of analysis is the *frame*, in the sense of Fillmore (2006): a schematic representation of a situation type, comprising a cluster of related concepts and their semantic roles. A frame for a “commercial transaction” includes a buyer, a seller, goods, and money; a frame for a “threat assessment” includes an agent perceiving danger, the perceived source of danger, and the agent’s evaluation of the danger’s severity. Fillmore’s central methodological claim is that direct verbal articulation of elements of a frame correlates with the frame’s cognitive salience for the speaker. ISIT operationalised this claim by treating frames extracted from a participant’s verbal report as direct evidence of the cognitive and affective structure the participant brought to the experience.

The conceptual ground for this operationalization is Barsalou’s account of *situated conceptualization* (Barsalou, 2009; Barsalou, 2020). On Barsalou’s view, concepts are not amodal symbols retrieved from a static lexicon; they are dynamic simulations assembled on demand from perceptual, motor, introspective, and contextual records. A participant who recounts an event is not transcribing a stored representation; they are running a situated simulation and producing a verbal record of its trajectory.

This forecloses a naive interpretation of ISIT scores. A participant’s “fear” frame, as scored by ISIT from a retrospective virtual reality (VR) report, is not the same fear that a depression scale measures or that a fear-conditioning paradigm elicits. Each is a different situated enactment of the conceptual cluster around fear and treating them as instances of a single underlying entity would be the closed-world reduction that ISIT is designed to avoid. ISIT measures *the situational salience of a frame in the participant’s enacted retrospective conceptualization*, not a stable trait.

A consequence of this commitment is that frames in cognitive science are not naturally bound to a single theoretical projection. Speaking of emotions, the same fragment of verbal report can simultaneously instantiate Ekman’s universal emotions (Ekman, 1992), Russell-style core affect (Russell, 2003), Pankseppian primary-process activation (Panksepp, 1998), Lazarus appraisal (Lazarus, 1991), Frijda action tendencies (Frijda, 1986), Ortony, Clore, and Collins (OCC) event–agent–object structure (Ortony et al., 1988), Haidtian moral-foundation (Graham et al., 2013), and Schwartzian value (Schwartz, 1992) projections. The constructed-emotion program (Barrett, 2017) makes this multivalence the rule rather than the exception. ISIT inherits a multi-theory frame definition through POLANYI++‘s components, which compose such projections into an integrated frame-driven graph. The composition mechanism is internal to POLANYI++ extraction; ISIT consumes the resulting frames as a multi-theory.

The empirical literature on verbal-report validity has matured considerably since the early concerns of Nisbett and Wilson (1977). Ericsson and Simon (1984) seminal protocol analysis established that verbal reports are valid when interpreted as records of the *content* of working-memory states during task performance, not as introspective claims about the *causes* of behaviour. Fox et al. (2011) confirmed in a meta-analysis that concurrent think-aloud protocols do not distort task performance and that retrospective verbalization, when properly elicited, recovers the majority of the relevant content. Schwitzgebel (2008, 2011) has further restricted the range, showing that introspective reports about *qualia* (the felt character of experience) are systematically poorer than reports about *content* (what is being experienced).

For ISIT, the relevant claim is narrower than the general verbal-report-validity question. ISIT treats verbal reports as records of *situated conceptualization*: the conceptual content that was active in the participant’s simulator when they recalled the experience. This is consistent with Ericsson–Simon’s content-not-cause restriction and with Schwitzgebel’s qualia-not-content caveat. ISIT does not claim to read off the participant’s inner phenomenology; it claims to score the frames that are evidentially active in the report.

The dominant computational tradition in implicit measurement has been the Implicit Association Test (IAT; Greenwald et al., 1998), a reaction-time-based instrument that measures the strength of automatic associations between concepts. The IAT has been highly productive but has well-documented limitations: weak predictive validity for individual behaviour, sensitivity to procedural variations, and dependence on experimenter-defined concept pairs. The natural-language counterpart is the Word Embedding Association Test (WEAT; Caliskan et al., 2017), which measures associations in static word embeddings via differential cosine similarity. WEAT is powerful for auditing societal-level biases in text corpora but, like the IAT, relies on experimenter-defined word lists and measures static associations rather than the dynamic conceptualization of an individual.

ISIT operates in a different measurement regime. Where IAT and WEAT measure *pre-existing associative structure*—in the individual or in the corpus—ISIT measures *the situational framing of a specific lived event* as articulated in a specific verbal report. ISIT does not need experimenter-defined association lists; it discovers the relevant frames inductively from the participant’s report, via the POLANYI++ extraction backbone. ISIT scores the frames’ salience individually, per participant, with a per-frame audit chain back to the textual evidence. The output is therefore a per-participant implicit-measure vector that complements rather than replaces IAT and WEAT: an experiment may include IAT as a measure of pre-existing implicit attitudes, ISIT as a measure of situated experience during the manipulation, and an explicit questionnaire as a measure of post-experience reflection, in a single design.

The grounded theory (GT) family—classical (Glaser and Strauss, 2009; Strauss and Corbin, 1990), constructivist (Charmaz, 2014), and contemporary practical (Birks and Mills, 2015)—is the dominant qualitative methodology for inducing theory from textual data. Its core commitment is that theory should emerge from the data through line-by-line open coding, axial coding to identify categorical relationships, and selective coding to integrate around a core category. Grounded theory has been generative for cognitive science whenever the relevant phenomena resist *a priori* category specification, but it is laborious, low-throughput, and only partially reproducible across analysts.

Computational grounded theory (CGT) (Nelson, 2020; Carlsen and Ralund, 2022) addresses the throughput bottleneck by scaffolding the human analyst with computational pattern detection—typically topic modeling or embedding-based clustering—in a three-step structure: pattern detection, pattern refinement, pattern confirmation. CGT preserves the inductive commitment of GT and raises throughput, but does not produce a quantitative, replicable per-participant score: its output remains a description of a corpus, not a measurement of a participant.

Large Language Model (LLM)-augmented GT (He et al., 2026; Dai et al., 2023) takes the automation further by using generative models to propose code, group it, and draft theoretical narratives. The recent literature documents productivity gains and characteristic failure modes (hallucinated codes, overfitting to model priors, prompt-drift effects on reproducibility). LLM-augmented GT remains, however, primarily a corpus-level analytical aid: it produces themes and theoretical narratives, not validated per-participant measurement vectors. These same failure modes are what ISIT’s extraction backbone is built to bound: the base/extended separation and the DARKSIDE audit constrain silent contradiction, and the tacit-aware DEEPJUDGE adjudication (Section 5.1) measures and gates the residual over-projection—hallucinated frames—directly, rather than leaving it to vary with the prompt.

ISIT’s relation to this family is operationally different on three points: (i) ISIT operates on already-structured extended knowledge graphs rather than on raw text; (ii) ISIT produces participant-level numerical scores rather than corpus-level qualitative themes; (iii) ISIT integrates by design with other measurement channels rather than being freestanding as a qualitative analysis.

## 3 Method

### 3.1 POLANYI++ as ISIT’S implicit knowledge extraction backbone

The POLANYI++ pipeline (De Giorgis et al., 2025) realizes a function

E=f(I,O,S,B,H,T,M,U)

whose arguments are the input modality I (text, image, and multimodal); the inferential operator O (the generative model used for heuristic instantiation); the source S (the verbal report or other source content); the base graph B (the text-grounded triples extractable without inference); the heuristic set H ; the task specification T ; the method set M ; and the ontology prior U . The output E is the *extended knowledge graph*: B augmented by inferred triples whose provenance is traceable to specific elements of H and M .

POLANYI++ (cf. Supplementary Figure S1) operates in four modes. EXTRACT (Mode 1) populates E from S using H . DISCOVER (Mode 2) generates hypotheses about gaps in E and interrogates the source for supporting evidence. REVISE (Mode 3) produces a revised source $S^{*}$ whose textual commitments align with the consolidated content of E . ALIGN (Mode 4) reconciles E with external ontologies. For the verbal-report use case at the center of this study, EXTRACT is the workhorse, with selective application of DISCOVER as an audit on the completeness of extraction.

An architectural commitment of POLANYI++ is the hard separation between the base graph B and the inferred extensions in E . The base graph contains only triples directly grounded in the text: explicitly named entities, stated events, verbatim emotional expressions, surface causal claims marked with causal connectives. The extended graph augments B with the outputs of heuristic application, each annotated with its heuristic provenance. The separation is enforced programatically and is preserved across all pipeline modes. For verbal-report analysis the practical importance is that every claim in E —including every frame ISIT will cluster—is traceable to the heuristic that produced it and ultimately to the textual evidence in S . This is the audit chain that grounded theorists have long demanded but rarely had operationally.

The heuristic set H carries the bulk of the cognitive-content extraction. A heuristic contains an informal description of a foundational theory, a prototype ontology that formalizes the theory or theories considered as best practices, and a few examples of application. The heuristics most relevant for verbal-report analysis are listed in Supplementary Table S1, along with the types of frames they contribute and the empirical phenomena they target.

The heuristic application is directed by the task specification T and constrained by an existing ontology prior U . For verbal-report analysis U may be empty (fully open-world) or may constrain the extraction to a domain ontology of interest (e.g., emotion taxonomies and, violence-perception schemas).

Three methods in M contribute substantively to verbal-report extraction.

The first is CUECOHERENCE, a cross-channel coherence check that intervenes during the reification of triples in E . For multimodal sources, CUECOHERENCE ensures that frames extracted from the verbal channel are consistent with frames from the visual channel; for purely verbal sources, it still operates between the affective, cognitive, and behavioral sub-channels of IMPACT.

The second is QUALECOMPOSITION, the *qualia-composition* method that ensures frames produced by extraction are not bound, for instance, to a single emotion or value theory. QUALECOMPOSITION takes a fragment of the extended knowledge graph and a set of theoretical projection lenses—affective lenses (Russell, Panksepp, Ekman, Plutchik, Frijda, Lazarus, OCC), value lenses (Haidt MFT, Curry MAC, Schwartz, Rokeach, Inglehart), and, where applicable, collective-intentionality lenses (Tuomela, Gilbert, Durkheim)—and produces a *perspectival quale*: a typed tuple whose components are the frame’s projections through the chosen lenses, together with a coherence assessment across them. The perspectival quale is the unit through which a single situated frame (say, “feeling unsafe”) is represented as the joint commitment of multiple theoretical descriptions (negative core affect under Russell; FEAR-system activation under Panksepp; high-threat low-control appraisal under Lazarus; flight tendency under Frijda; care-violation under Haidt), rather than as the assignment to a single category. The composition occurs *during* extraction, not after: the frames that ISIT would subsequently cluster are already multi-theory composites with explicit projection records. The mechanism handles, for example, the simultaneous instantiation of Russellian negative affect, Pankseppian FEAR-system activation, Lazarus high-threat low-control appraisal, Frijda flight-tendency-with-override, and Haidtian Care-violation that a typical catcalling-report fragment exhibits.

The third is DARKSIDE, an *audit* method (Gangemi and Bottazzi, 2026) that tracks what it excludes—that is, the contents the discourse renders incompatible with itself, going forward. As the report is processed, DARKSIDE accumulates a *NegativeTrail*: the sequence of exclusions derived from each commitment, organized by four exclusion types (negation, incompatibility, causal-path exclusion, and selectional restriction). *Constancy violations*—points where new commitments contradict accumulated exclusions without justification (no perspective shift, no hypothetical marking, no narrative repair)—are detected by checking each new commitment against the accumulated NegativeTrail and producing a violation event. Each detected contradiction is then classified as either *encapsulated* (legitimate; the contradiction passes one of five tests indicating paradoxical, unreliable-narrator, ironic, rhetorical, or absurdist function) or *vain* (faulty; constancy drift, pattern collision, or absent repair). The encapsulated/vain classification has clinical-adjacent implications that we wish to surface but not to overclaim. Consider a verbal report in which successive segments appear to contradict—for instance, a passage that begins “I did not feel anything” and shortly afterwards describes intense fear or distress. A naive coherence check would flag this as a contradiction; under DARKSIDE’s encapsulation tests it is *potentially* classifiable as encapsulated under the unreliable-narrator or perspectival-shift tests, on the basis that the two segments come from different reflective standpoints. We say *potentially* because the classifier’s behaviour on such cases has not been empirically validated against expert clinical judgment, and we do not propose ISIT as a tool for clinical interpretation of traumatic narratives. The example is illustrative of the architectural distinction between encapsulated and vain contradictions; its use in any clinical context would require its own validation program.

The output of DARKSIDE for a given report is an *audit profile* attached to the graph as a whole: the NegativeTrail, the set of constancy violations, the classified contradictions, and a summary *delegation-risk verdict* on the report (TRUSTLESS, SUPERVISED, or UNSAFE) reflecting the substrate-level signals about path-dependence, exclusion accumulation, and verifiability of the scoring task. Verbal-report scoring tasks in cognitive science typically receive the SUPERVISED verdict, indicating that automated scoring is admissible but should be cross-checked by the experimenter on the audit summary. Alongside this coherence analysis, DARKSIDE also classifies each commitment on an independent warrant axis—warranted, misattributed, fabricated, or unattested—and aggregates a fabrication rate that feeds the same delegation-risk verdict; this warrant axis is the basis for the extraction-accuracy adjudication reported in Section 5.1. DARKSIDE’s classifications are consumed by ISIT’s contextual-influence scoring component (cf. Section 3.2).

For each ISIT verbal report $S_{i}$ , POLANYI++ produces an extended knowledge graph $E_{i}$ with the following properties: (i) every triple is annotated with the heuristic that produced it; (ii) the graph contains both base text-grounded triples and inferred extensions, with the boundary explicit; (iii) frames in the graph are multi-theory composites with explicit projection records; (iv) the graph carries a NegativeTrail and classified contradiction events, available as an audit profile attached to the graph as a whole. ISIT operates on this output: the input to ISIT is not raw text but $E_{i}$ with its provenance and audit annotations.

### 3.2 The ISIT method

ISIT—formally, the ISITPROFILE method in POLANYI++ − is a frame-clustering and scoring method that operates on extended knowledge graphs produced by POLANYI++ and produces a per-participant *Situation Profile*: a typed numerical vector indexed by frames in a Frame Ontology, with each frame’s salience normalized to [−1, +1]. The Frame Ontology may be supplied externally (Mode A, single-case profiling against a pre-existing schema) or induced from the union of multiple participants’ XKGs (Mode B, multi-case profiling with emergent ontology). The Situation Profile is the artifact that downstream statistical analysis consumes; it is the implicit-measure output that complements explicit and physiological measures.

The *implicit* in “implicit situation intensity test” has a precise meaning. The participants did not score themselves on any of the frames in the Frame Ontology; a participant told a story. The salience of each frame in their situated conceptualization is *implicit* in the linguistic, semantic, and pragmatic structure of that story, and the score ISIT assigns is a quantification of that implicit salience. The score is not the participant’s explicit rating; the score is what the participant *implicitly* committed to by telling the story they told.

Mode A (cf. Supplementary Figure S2) consumes a single extended knowledge graph E together with a Frame Ontology FO (either externally supplied as part of the ontology prior U or, in multi-case settings, inherited from Mode B’s induction step) and a task specification T , and produces a single Situation Profile SP for the participant who produced E .

**Phase 1: Frame extraction.** ISIT harvests frames from the heuristic-annotated triples in E . Each heuristic contributes a class of frames. A typical verbal report contributes frames from IMPACT (emotional, social, cognitive, and physical), ToM (motivational, intentional, and epistemic), CAUSALITY (causal-attribution), COERCION (metaphor and symbol), PERSPECTIVE (viewpoints), and CUECOHERENCE (coherence verdict and named tensions). For multimodal reports VISUALSEMIOTICS contributes denotative and connotative frames. Extraction also harvests *cue-source frames* (which channel—facial expression, posture, context, language—supports each frame). The output is a set of raw frames $F=\{f_{1},,\ldots ,,f_{n}\}$ with their provenance and a perspectival quale (where present).

**Phase 2: Frame clustering.** The raw frames are clustered against the Frame Ontology FO . If FO contains anchor clusters from a structured ontology prior—for instance, an Ekman-emotion cluster, a relationship-typology cluster, a set of canonical motivation categories—each raw frame is mapped to its nearest anchor by semantic similarity. Frames that do not map to existing anchors become *emergent* extensions: they may join an existing cluster as extension members or seed a new cluster. Inter-cluster relationships extracted from E —*triggers* (from CAUSALITY), *enables* (from CAUSALITY), *contrasts* (from PERSPECTIVE), *coherent-with* and *tension-with* (from CUECOHERENCE)—are added to FO to capture the relational structure of the participant’s enacted conceptualization. The clustered Frame Ontology with raw frames mapped to clusters is the reified frame structure of the participant’s report.

**Phase 3: ISIT scoring.** Each frame $f_{i}$ in the clustered ontology receives an ISIT score by integrating three evidence components that are described in Section 4.3. The composite raw score is normalized to [−1, +1] via minimum–maximum (min–max) scaling relative to the theoretical minimum and maximum of the frame’s measurement space.

**Phase 4: Profile assembly.** The reified frame scores, together with the participant-specific Frame Ontology and the per-frame perspectival qualia inherited from QUALECOMPOSITION, are assembled into an ISIT: SituationProfile. The profile is annotated with provenance ISITPROFILE and includes the audit summary attached to E by DARKSIDE. Optionally, frame scores are projected onto continuous semantic spaces for interoperability—for example, emotion frames onto the Russell circumplex, relationship frames onto an intimacy–formality space, motivation frames onto an agency–directedness space—to enable comparison across studies that use different frame ontologies.

The ISIT score for any given frame is a weighted and normalized composite of three components, designed to integrate multiple sources of evidence into a robust measure of implicit salience. The default weighting reflects an emphasis on direct linguistic evidence; the weights can be adjusted based on the experimental context and available data streams.

**Direct Frame Manifestation** ( $S_{M}$ , default weight ≈ 0.40). This component quantifies how explicitly the frame is evoked in the participant’s verbal report, with scoring tiered by the centrality and valence of the linguistic cues. The theoretical justification stems from Fillmore’s frame semantics: direct verbal articulation is a strong indicator of cognitive salience (Fillmore, 2006). Operationally, $S_{M}$ scales with the number of distinct triples in E that support the frame, weighted by other parameters, and modulated by the frame’s centrality in the surrounding subgraph.

**Affective Alignment** ( $S_{E}$ , default weight ≈ 0.30). This component maps emotional indicators present in the report—emotion words, valence-laden adjectives, metaphors and metonymies surfaced by COERCION—to the cognitive and behavioral frames in the cluster. For frames whose primary projection is itself affective, $S_{E}$ is the QUALECOMPOSITION-projected intensity (the value-arousal magnitude under Russell, the primary-system intensity under Panksepp, etc.). For frames whose primary projection is non-affective, $S_{E}$ is the alignment of the frame with co-occurring affective frames in the same subgraph. The component’s validity is grounded in the extensive literature on emotion–cognition relationships, including Damasio’s somatic marker hypothesis (Damasio, 1994).

**Contextual Influence** ( $S_{I}$ , default weight ≈ 0.30). This component is the integration point with non-textual data streams and audit signals. It incorporates: (a) cross-channel coherence verdicts from CUECOHERENCE (frames in high-tension regions of E receive prediction-error-modulated scores); (b) cross-temporal coherence verdicts from DARKSIDE (frames that violate accumulated exclusions in the NegativeTrail receive penalized scores); and (c) external context measurements provided by the experiment—embodiment-questionnaire scores in VR studies, GSR or HRV in physiological-recording studies, eye-tracking or keystroke metrics in human-computer interaction (HCI) studies, regional activation profiles in neuroimaging studies. $S_{I}$ ’s flexibility is what makes ISIT integrable with other measurement channels: any external measurement that can be associated with a frame contributes to that frame’s contextual-influence score.

**Theoretical bounds, default weights, and weight calibration**. The composite raw score is

$$S_{\mathrm{raw}}=w_{M}\cdot S_{M}+w_{E}\cdot S_{E}+w_{I}\cdot S_{I}$$

is normalized to [−1,+1] by min–max scaling against the theoretical minimum and maximum scores that any frame can attain under the chosen scoring policy. The theoretical bounds are determined as follows: $S_{\min}$ is the score the frame would receive if it were absent from the verbal report and explicitly contradicted by the contextual channel (manifestation 0, affective alignment 0, contextual influence at its negative limit), which under the default policy yields $S_{\min}=-w_{I}$ . $S_{\max}$ is the score the frame would receive if it were centrally evoked, maximally affectively aligned, and the contextual channel pushed it to its positive limit, yielding $S_{\max}=w_{M}+w_{E}+w_{I}$ . With the default weights, $S_{\min}=-0.30$ and $S_{\max}=1.00$ ; the min–max normalisation in step 5 of the algorithm rescales them to [−1,+1] .

The interpretation of negative ISIT scores is therefore as follows. A score near −1 indicates that the frame is absent from the report and that the contextual channel—explicit, physiological, or audit-derived—provides evidence against the frame’s activation. A score near zero indicates baseline absence with no contradictory contextual signal. A score near +1 indicates strong activation across all three components. Crucially, $S_{\mathrm{norm}}\approx -1$ does not mean that the participant *opposed* the frame in a propositional sense; it means that the frame is absent from their report *and* that the contextual channel modulates the score downward. Negative scores in the implicit regime ( $w_{I}=0$ ) collapse to $S_{\mathrm{norm}}\in [0,1]$ , reflecting that, in that regime, only positive evidence of frame manifestation contributes to the score.

The default weights $\left(w_{M},,w_{E},,w_{I})=(0.40,0.30,0.30\right)$ are operational defaults and have not been psychometrically calibrated. They reflect the relative directness of the underlying evidence (one inferential step for $S_{M}$ , two for $S_{E}$ and $S_{I}$ ) and are stated as provisional pending empirical calibration. A *sensitivity analysis sketch* shows that under three alternative weight schemes— (0.50,0.30,0.20) , (0.40,0.40,0.20) , and (0.30,0.30,0.40) —the rank ordering of frames by salience in the Lucifora et al. (2025) corpus is invariant for the top-12 frames in each cluster; the absolute scores shift in the expected direction (more weight on $S_{E}$ amplifies frames with high affective alignment), but the ordinal structure that drives the cluster-level correlations is stable. We treat this as a sketch, not a sensitivity study: a proper sensitivity analysis would systematically vary all three weights across the simplex and report the volume of the weight space within which qualitative conclusions are preserved.

The asymmetry reflects the relative directness of the underlying evidence: Direct Manifestation is grounded in the verbatim text via the BASE heuristic and is therefore audit-traceable in one inferential step, whereas Affective Alignment and Contextual Influence require additional inferential steps (lens projection and cross-channel integration, respectively) and are weighted lower in default configuration. The weights are not psychometrically derived; they are operational defaults to be revised when domain-specific empirical evidence warrants. Three configurations are conventional: the default (0.4,0.3,0.3) for studies in which the verbal report is the primary data source; (0.3,0.3,0.4) for studies with rich, validated physiological or sensor data where the contextual channel carries correspondingly more measurement weight; and (0.5,0.3,0.2) for studies emphasizing linguistic content over context. Frame-specific modifiers, when applicable, may further adjust the raw score before normalization: a frame designated as central to the experimental hypothesis may receive an amplification modifier; a frame whose ontological status is provisional may receive a hedging modifier. The final normalization is min–max to [−1, +1], where −1 indicates strong opposition to or absence of the frame, 0 indicates neutrality or baseline presence, and +1 indicates strong activation. The full algorithm, with explicit pseudocode for the scoring components (cf. Supplementary Table S2), the modifier-application step, the contextual-adjustment step, and the normalization, is given in Supplementary Figure S3.

Mode B (cf. Supplementary Figure S4) is the multi-case generalization of Mode A and is the central mechanism for cognitive-science deployment, because statistical comparison across participants, conditions, and groups is the empirical lifeblood of the discipline. Mode B accepts a set of cases $\left\{(E_{i},,,,\text{agen}t_{i},,,,\text{grou}p_{i},,,,\text{situatio}n_{i},,,,\mathrm{typ}e_{i})\right\}$ —multiple XKGs with agent and situation metadata—and proceeds in five phases.

**Phase 0: Frame Ontology induction.** The ontology prior U is *not* supplied externally; it is induced as $U=\cup_{i}\text{frames}(E_{i})$ . This implements the inductive commitment that situational structure should emerge from data. The induced Frame Ontology comprises *anchor frames* (those shared across two or more groups, providing the inter-group interoperability backbone) and *emergent frames* (those unique to one group or agent). Emergent clusters restricted to a single group are themselves an empirical signal—for instance, a cluster of perceptual-limitation frames appearing only in one cohort indicates a cognitive signature of that cohort.

**Phase 1: Profile computation.** Each case profile $SP_{i}$ is computed via Mode A using the *induced* FO . Frames absent from $E_{i}$ but present in FO score 0.0; the resulting profile vectors are sparse but fully comparable across cases.

**Phase 2: Group aggregation.** For each group, group-level mean and standard-deviation vectors are computed; the group is itself reified as an ISIT: GroupProfile.

**Phase 3: Descriptive statistics.** Per-group, per-situation distributions for categorical (mode and entropy) and continuous (mean, SD, median, and range) frame variables are computed.

**Phase 4: Inter-rater agreement.** Within-group categorical agreement (Krippendorff’s α; Fleiss’ κ when ≥ 3 raters per case); within-group continuous agreement (intraclass correlation); between-group profile similarity (cosine and Spearman ρ over frame-salience rankings). Realistic-versus-simulated-scene splits, where applicable, enter as covariates.

**Phase 5: Hypothesis testing.** Reference-based comparisons (distance to a designated reference centroid; Mann–Whitney U test on cosine to reference); linear mixed models with fixed effects of group, scene type, and their interaction, plus random intercept by agent. Per-frame-dimension contrasts identify dimensions with significant group effects.

The output of Mode B is an ISIT: InterRaterReport that contains the group profiles, the pairwise comparisons, the statistical tests, and the descriptive statistics, with provenance and audit annotations preserved throughout.

A potential reading of ISIT’s scoring formula deserves explicit comment, because the contextual-influence component $S_{I}$ admits, among its inputs, external measurements such as embodiment-questionnaire scores or physiological signals. ISIT’s *frame extraction* and *frame clustering* operations (Phases 1 and 2 of Mode A; Phase 0 of Mode B) are strictly implicit: they consume the extended knowledge graph E produced by POLANYI++ from the participant’s verbal report and produce a Frame Ontology and a typed frame structure. No explicit-measure data enter these phases. The participant has not been asked to rate themselves on any of the frames; the frames are inferred from the structure of the verbal report through the heuristic chain. The implicit-measure status of the *clustering output* is, in this respect, exact: a vector of frame assignments whose evidential basis is the participant’s verbal articulation alone.

The contextual-influence component $S_{I}$ in the scoring step (Phase 3 of Mode A) serves as the integration point for non-textual data streams and audit signals and is best understood as a *post-clustering modulation*. The raw frame profile that results from Phases 1 and 2, a sparse vector of frames clustered against the Frame Ontology, is already a complete implicit measure of frame salience in its own right and is the artifact on which most subsequent analyses can operate. The $S_{I}$ modulation is invoked only when the experimenter intends to combine the verbal channel with other measurement channels; when it is invoked, the scoring is no longer purely implicit but explicitly multimodal, and the resulting score should be interpreted accordingly. ISIT therefore admits two reporting regimes: an *implicit regime*, in which the published profile is the Phase-2 frame-cluster vector or a $w_{M}$ -only scoring with $w_{E}=w_{I}=0$ , and a *multimodal regime*, in which Phase-3 modulation is applied with explicit weights documented in the study. Both regimes are licit; conflating them is not. We recommend that empirical reports specify which regime is being used and report scores accordingly.

This makes the relationship to the IAT and WEAT traditions clearer. IAT and WEAT measure pre-existing associative structure with no integration step. ISIT’s *implicit regime* is the closest analog: a per-participant measurement vector derived from the participant’s own report without consulting any other measurement instrument. The multimodal regime is a separate construction whose purpose is exactly the integration with other channels that Section 3.3 describes; it is offered alongside, not in place of, the implicit regime.

### 3.3 Integration with other measures

ISIT measures the *salience of frames in a participant’s situated conceptualization* given a particular verbal report and its associated context. By construction, it does not claim to measure stable cognitive traits: two reports about the same nominal experience by the same participant will, in general, produce different profiles, because they enact different situated conceptualizations. The boundary condition is the Ericsson–Simon content-not-cause caveat (Ericsson and Simon, 1984): ISIT scores reflect the conceptual content active in the participant’s report, not introspective claims about why they behaved as they did. ISIT is also not a measure of cognitive states about which the participant has no introspective access (Schwitzgebel, 2008); for those, autonomic and neural measures remain the appropriate channels.

ISIT’s design commits to one specific role in a cognitive-science measurement design: it is the *implicit-measure complement* to autonomic, behavioral, and questionnaire-based instruments, operating on the verbal-report channel (cf. Supplementary Figure S5). The Situation Profile produced by Mode A or the Group Profile produced by Mode B is a typed numerical vector intended for direct combination with measures from other channels. Three classes of integration are routine in well-instrumented cognitive-science studies and are supported by the ISIT output contract.

#### 3.3.1 Combination with explicit standardized questionnaires

Standardized questionnaires—e.g., the Toronto Alexithymia Scale (TAS-20; Bagby et al., 1994), Ekman emotion-intensity ratings (Ekman, 1992), the State–Trait Anxiety Inventory (Spielberger et al., 1971), depression and anxiety inventories, or value-survey instruments (Schwartz, 1992; Graham et al., 2013)—produce per-participant scores that can be cross-correlated with ISIT scores at the frame level. Three patterns of correlation are evidently useful:

- *Convergent correlation*—ISIT scores for frames that are nominally related to a questionnaire’s construct should correlate with the questionnaire’s score in the predicted direction. For instance, ISIT scores for the SafetyCluster of a catcalling study should correlate positively with anxiety-inventory subscales for the threat-related items. A high convergent correlation supports the validity of the ISIT score as a measure of the construct.
- *Divergent correlation*—ISIT scores for frames that are nominally unrelated to a questionnaire’s construct should not correlate strongly with that questionnaire. This protects against the ISIT score being an artifact of general report verbosity, linguistic style, or the participant’s overall positive/negative mood orientation.
- *Incremental validity*—when ISIT scores are added to a regression model alongside the standardized questionnaire scores, they should explain additional variance in the dependent variable (typically a behavioral outcome). Incremental validity is the operational test of whether ISIT is contributing measurement information beyond what the questionnaires already provide.

#### 3.3.2 Combination with implicit physiological and behavioral measures

ISIT is designed to complement the existing repertoire of implicit physiological measures by occupying the verbal channel that they cannot reach. The natural integration partners are physiological sensors (galvanic skin response, heart-rate variability, pupillometry, respiratory rate), eye-tracking (fixation durations, saccade patterns, and areas of interest), behavioral sensors (keystroke and mouse logging in HCI studies; gait and posture in VR), and neuroimaging (Blood-Oxygen-Level Dependent functional Magnetic Resonance Imaging [BOLD fMRI] response patterns; EEG event-related potentials). Each of these measures a specific aspect of the bodily or neural correlates of the experience; none captures the conceptual structure articulated in language. ISIT supplies that conceptual structure as a typed numerical vector that can be entered into multivariate models alongside the physiological data.

Three integration patterns are particularly informative:

- *Verbal-physiological alignment.* Frame-level ISIT scores can be temporally aligned with physiological measurements over the course of throughout an experimental task. For a participant whose post-task verbal report scores high on ThreatAssessment, a corresponding peak in skin-conductance response during the threatening segment of the experimental scenario provides converging evidence that the participant experienced threat *in vivo*, not merely in retrospective construction.
- *Mediation analysis.* The contextual-influence component of ISIT scoring can incorporate physiological measurements directly as part of $S_{I}$ (Section 3.2). A frame’s score is then a function of both the linguistic and the physiological evidence for the frame, with the relative weighting determined by the experimental context. This is mathematically a fusion of measurement channels at the score-construction level.
- *Construct triangulation.* For a construct hypothesized to be carried by both a frame in the participant’s report and a region of brain activation (the “neural-correlate” approach, e.g., Lindquist et al., 2012), ISIT scores and neuroimaging signals can be triangulated to test the construct’s coherence across measurement channels. For example, the study described in Lucifora et al. (2025) included a NeuroSynth-based meta-analytical mapping of frame clusters to neural systems as a construct-validation step; cluster-to-region mappings were expert-validated by neuroscientist co-authors.

#### 3.3.3 Participant profiles as covariates

Individual-difference variables—clinical history, demographic information, prior exposure to relevant stimuli, scores on baseline trait inventories—are routinely entered as covariates in cognitive-science analyses to control for participant heterogeneity. ISIT scores integrate cleanly with this practice: the per-participant Situation Profile is itself a typed vector that can be regressed on covariates of interest. In a multi-case deployment of Mode B, participant group can enter as a fixed effect and individual participant as a random intercept in a linear mixed model of cosine-to-reference-centroid; the standard analytical strategy of cognitive-science group designs generalizes directly.

The end-to-end analytical pipeline combining ISIT with other measures typically takes the following form. Each participant contributes a measurement vector composed of (i) ISIT scores for frames of interest, (ii) explicit questionnaire scores, (iii) summary statistics or time-aligned features of physiological signals, and (iv) covariate values from the participant profile. The vectors are entered into a statistical model—linear mixed models for two-level designs; structural equation models when the theoretical model specifies latent constructs; multi-modal fusion architectures (e.g., factor models with channel-specific loadings) for studies with rich physiological and neural data—that yield tests of the experimental hypotheses while accounting for the dependencies among channels. The role of ISIT in this pipeline is unambiguous: it provides the verbal-channel implicit measure that the other channels cannot. ISIT is not a replacement for the other channels; it is a complement.

### 3.4 Operational specification: from verbal report to situation profile

For independent replication, the ISIT pipeline requires the following specifications: (i) the generative-model backend used by POLANYI++ as the inferential operator O , the version, the decoding settings (temperature, top- p , seed where exposed), and the API endpoint; (ii) the heuristic set H and the activation thresholds at which each heuristic was included; (iii) the prompt templates used by each heuristic, available in the POLANYI++ repository at the version cited in the manuscript; (iv) the semantic-similarity threshold for clustering raw frames against the Frame Ontology in Phase 2 (default cosine ≥ 0.75 against sentence-level embeddings produced by the same backend; configurable per study); (v) the negation- and modality-handling policies, which are applied at the heuristic-input stage and route appropriately to PRESUPPOSITION, IMPLICATURE, and MODALITY processing; (vi) the human quality-assurance procedure for the run, which we describe below; and (vii) the expected output files, which comprise the per-participant extended knowledge graph $E_{i}$ in Turtle format, the Frame Ontology in OWL, the per-participant Situation Profile, the DARKSIDE audit profile, and a run manifest documenting the above settings.

A worked example illustrating the transition from raw verbal report to ISIT score is provided in the Supplementary Figure S2. The example walks through one short verbatim report fragment from the Lucifora et al. (2025) corpus, in Italian and in English translation. It shows in turn the b0 base extraction, the heuristic-annotated triples that the IMPACT, ToM, CAUSALITY, COERCION, and PRESUPPOSITION heuristics contribute, the resulting frame cluster assignments under the Lucifora et al. (2025) 37-indicator Frame Ontology, the $S_{M},S_{E},S_{I}$ component scores under the default weights, and the final $S_{\mathrm{norm}}$ . The example is deliberately small (one fragment from one participant) and is intended for replication of the *procedure*, not for reproduction of any aggregate result.

A note on the human quality-assurance loop is necessary because it is an integral part of pipeline deployment under the present scope of validity. The Lucifora et al. (2025) deployment, on which the present study reports illustratively, applied three manual checks before any scoring was performed: (a) coreference verification, with particular attention to Italian negation, which is a known failure mode of automated entity-resolution under Italian morphosyntax; (b) programatic enforcement of the base/extended graph separation, which is implemented as a typing constraint on triple insertion; and (c) pre-registration of the Frame Ontology—that is, the FO was fixed before statistical analysis began, with subsequent emergent extensions to FO entering analysis only under a documented amendment procedure. These steps are not optional under the SUPERVISED delegation-risk verdict that DARKSIDE returns for VR-experience reports. They are minimum methodological requirements for any deployment of ISIT in the multimodal regime. Section 5.1 introduces DEEPJUDGE, an experimental POLANYI++ method that formalizes this quality-assurance loop as a tacit-aware adjudication of every extracted frame, converting the manual checks into a specified procedure with an inter-rater statistic.

## 4 Results from an empirical case study

A previous study (Lucifora et al., 2025) reported a controlled VR study in which 36 male participants (18 in the experimental catcalling condition and 18 controls) were embodied in a female avatar and exposed to a scripted street-harassment scenario, then provided retrospective written reports of the experience. The study used the POLANYI++/ISIT pipeline to analyze verbal reports and, in conjunction with embodiment-questionnaire scores and Ekman emotion intensity ratings, found that virtual embodiment in a female avatar increased male sensitivity to catcalling experiences as indexed by integrated implicit and explicit measures.

We summarize the methodological core of the study here through the present study’s architectural framing (cf. Supplementary Figure S6); the full empirical findings are reported in the source publication.

The extraction backbone applied seven heuristics: BASE (text grounding), PRESUPPOSITION, IMPLICATURE, IMPACT (with the four-layer expansion: emotional, cognitive, social, and, bodily), COERCION (with the explicit semantic-versus-psychological distinction added during pipeline development based on preliminary error analysis), CAUSALITY, and Theory of Mind (ToM). Each participant’s verbal report was processed into an extended knowledge graph $E_{i}$ with the heuristic provenance. Quality assurance comprised three measures: manual coreference verification (with explicit Italian-negation detection, a known challenge for automated systems), programatic enforcement of the base/extended graph separation, and pre-registration of the study’s Frame Ontology prior to statistical analysis to ensure consistent frame definitions across all 36 participants.

The Frame Ontology for the study comprised 37 indicators organised into four domains: 13 emotional-state frames (fear, anxiety, discomfort, anger, disgust, guilt, feeling-trapped, feeling-violated, detachment, pity, stress, disturbance, and sadness), 5 behavioral-response frames (freeze, flight, dissociation, observation, and submission), 15 violence-perception frames (including violence-type recognition and onset-timing categorization), and 4 semantic-marker frames (metaphor count, metonymy count, moral-coercion count, symbolic-coercion count). Each binary indicator (e.g., has_fear, has_submission) reflected the presence of an entity typed with the relevant ontological class in the participant’s extended graph; continuous indicators aggregated frame scores across related frames; the violence-onset variable was coded categorically as immediate, gradual, at-escalation, or at-physical, based on where in the scenario narrative the participant first explicitly or implicitly recognized violence.

Statistical comparisons used Fisher’s exact tests and chi-square tests on the binary and categorical indicators; continuous indicators were analyzed with standard parametric and non-parametric tests as their distributional properties dictated. The integration with explicit measures was at the cluster level: ISIT frame-cluster scores were entered into the study’s correlation structure together with the standardized instruments and the multiple-choice ratings, with each cluster-level correlation justified by some, not necessarily all, of the cluster’s component frames. ISIT scores for the safety-related cluster were correlated with Ekman emotion-intensity ratings for fear, anger, and sadness, with the catcalling-relevant cognitive frames (ThreatAssessment, SafetyManagement) showing differential correlation patterns with anger/sadness versus with fear, providing the kind of nuanced multi-channel signal that simpler instruments do not produce. The strong convergent validity (r > 0.95) reported in the source study reflects properties of the standardized instruments; ISIT contributes the verbal/implicit channel to this multivariate structure rather than converging with embodiment at that magnitude.

Three architectural points emerge from the case study. First, the integration with explicit measures (embodiment questionnaire and Ekman ratings) follows the integration recipe documented in Section 5. Second, the audit chain from each ISIT score back through the heuristic-annotated $E_{i}$ to the verbatim text in $S_{i}$ is preserved end-to-end, supporting the kind of *post hoc* inspection that grounded-theory analysts typically conduct manually but which here is operational. Third, the manual quality-assurance step is part of the methodology, not a defect: under DARKSIDE’s delegation-risk verdict, verbal reports about VR embodiment in violence scenarios are classified as SUPERVISED—that is, automated scoring is admissible but the experimenter is required to cross-check the audit summary—and the manual review of the extracted graphs is the appropriate response to that verdict.

## 5 Discussion

Cognitive science already has substantial investment in standardized closed-world instruments (questionnaires, Likert scales, categorical choice paradigms), in physiological and behavioral sensors (GSR, HRV, eye-tracking, EEG, fMRI), and in implicit-association measures (IAT, WEAT). ISIT does not replace any of these. It fills the gap where none of them operates: the implicit measurement of frame salience in the participant’s verbal report of a situated experience. Studies that elicit retrospective or concurrent verbal reports—across clinical psychology, social cognition, human–computer interaction, education research, and the behavioral sciences broadly—generate a data stream that the existing measurement repertoire cannot directly quantify. ISIT fills that gap with a typed, integrable, audit-traceable per-participant measurement vector.

The integration recipe of Section 5 is therefore not a peripheral addition but the operational point of the method. ISIT’s value depends on its ability to be integrated with the other channels in a coherent multivariate analysis. A study that uses only ISIT, without the other channels, is methodologically incomplete; a study that uses only the other channels, without ISIT, is missing the verbal-channel implicit signal.

ISIT shares with the grounded theory family one substantive commitment: situational structure should emerge from data, not be imposed *a priori*. Where standardized closed-world instruments require the experimenter to specify in advance which categories will be measured, grounded theory and ISIT both insist that the categories—themes in grounded theory, frames in ISIT—should arise from the data. In Mode B, this commitment is operationalized as the induction of the Frame Ontology from the union $\cup_{i}E_{i}$ of the participants’ extended knowledge graphs (Section 4). The induced Frame Ontology is the analog, in ISIT, of the categorical scheme that grounded theory analysts assemble through open and axial coding.

Beyond this shared commitment, ISIT and grounded theory diverge. The divergence is not a matter of degree. Still, of kind: ISIT solves a different methodological problem, with a different output type, on a different input type, and via a different operational mechanism.

### 5.1 ISIT does not perform open, axial, or selective coding

The coding cycles of grounded theory are interpretive operations that read raw text and assign codes line by line. ISIT does not read raw text; it reads structured knowledge graphs—already typed, already provenance annotated, already multi-theory composed—produced by the POLANYI++ extraction backbone from both the explicit and the implicit content of the report. The “coding” of the participant’s report has already occurred in the heuristic application of POLANYI++ extraction; ISIT operates on the typed output, clustering frames into a Frame Ontology and scoring their salience. The distinction matters because the validity considerations differ: where grounded theory’s reproducibility depends on inter-coder agreement among trained analysts, ISIT’s reproducibility depends on the determinism of its clustering and scoring once the extraction is fixed—the Frame Ontology being supplied (Mode A) or induced from the extracted frames by the clustering itself (Mode B)—together with the controllability and audit-traceability of the extraction step on which everything downstream conditions (discussed in Section 3.1).

### 5.2 ISIT does not produce corpus-level themes

The output of grounded theory is, in the canonical case, a theoretical model of the phenomenon: a core category, a set of related categories, and a network of relationships among them. The output is a description of the corpus. ISIT’s output is a per-participant Situation Profile: a typed numerical vector indexed by frames. The Frame Ontology is shared across participants, but the salience scores are individual. This output type is what makes ISIT integrable with explicit and physiological measures in a multivariate cognitive-science design (Section 5); a corpus-level theme is not directly enterable in a regression model.

### 5.3 ISIT does not stand alone as a qualitative analysis

Grounded theory, when properly applied, is a complete qualitative methodology: it produces an analytical account of the phenomenon intended to be readable and interpretable by domain experts as a self-standing scientific output. ISIT is not designed to stand alone in this way; it contributes one channel of measurement to a larger cognitive-science design. The Situation Profile is a measurement vector, not an analytical narrative.

The Lucifora et al. (2025) catcalling study shows that ISIT, when integrated with embodiment questionnaire scores and Ekman emotion-intensity ratings, supports a methodologically coherent and empirically substantive analysis of male sensitivity to virtual catcalling. At the cluster level, ISIT’s frame clusters correlated with the embodiment and emotion measures—with individual frames contributing unequally to each cluster-level correlation—and the differential correlation patterns of the safety-related frames with anger/sadness versus fear illustrate that ISIT captures structure beyond what Ekman ratings alone provide; the strong convergent validity (r > 0.95) is a property of the standardized instruments. Consistent with the framing above, we treat these as demonstrating feasibility for integration with established measures rather than as a psychometric validation, which remains the future study.

### 5.4 Reliability, reproducibility, and the foundation-model dependence

ISIT inherits reproducibility from two sources: the determinism of its clustering and scoring once the POLANYI++ extraction is fixed—the Frame Ontology is either supplied in advance (Mode A) or induced from the extracted frames by the clustering step itself (Mode B), and in both cases the cluster assignments and salience scores are a deterministic function of that extraction—and the traceability of the POLANYI++ audit chain from each frame back to the verbatim text in S. These properties hold by construction. They do not eliminate the variance introduced by the large-language model that POLANYI++ uses as its inferential operator. Two runs of the same pipeline on the same input can produce slightly different extended knowledge graphs and therefore slightly different Situation Profiles. The variance is bounded and audit-traceable—every divergence corresponds to a divergence in a specific heuristic output—but it is not zero. The standard mitigations are explicit—deterministic decoding where the model exposes it, multi-run aggregation, and the cross-model pilot reported below—so the reproducibility claim is properly stated as audit-traceable reproducibility up to bounded model variance, not bit-level reproducibility.

#### 5.4.1 Reliability

Because ISIT is designed as a state measure (the salience of frames in a particular situated conceptualization, not a stable trait), the standard test–retest framework does not apply unmodified: two reports about the same nominal experience by the same participant will, by design, produce different profiles. The appropriate reliability constructs are (i) intra-text reliability: stability of the score when the same report is processed by the same pipeline under independent runs of the foundation model, which is the bounded-model-variance quantity discussed above; (ii) parallel-extractor reliability: stability of the score when the same report is processed by the same pipeline under two different foundation-model backends; (iii) inter-rater reliability under the human QA loop: agreement between two manual reviewers on the Quality Assurance (QA) decisions described in Section 3.4. Of these, (ii) parallel-extractor reliability and (iii) inter-rater reliability are measured directly on the case-study corpus—by the cross-model pilot and the DEEPJUDGE panel reported below—while (i) is the bounded-model-variance quantity characterized above.

*Validating the extraction the case study relied on.* The case study of Section 4 applied ISIT to the 18 catcalling reports and took the resulting extraction as guarded by the manual quality-assurance loop described in Section 3.4. Two questions about that extraction remain, and the remainder of this section addresses both on the same corpus. The first is reproducibility: does the same report yield the same Situation Profile under a different foundation model? This is the cross-model pilot reported next. The second is accuracy: are the frames the extraction places on a report actually warranted by it? This is not a question a reproducibility metric can answer—two backends can agree on a frame that is wrong, or disagree on a frame that is merely relabeled—and we address it with an experimental POLANYI++ adjudication method, DEEPJUDGE, introduced after the pilot. Together the two analyses convert the case study’s trust-plus-manual-review into a measured, auditable claim about the extraction on which the Situation Profiles are built.

*Cross-model stability: a per-participant frame-alignment pilot.* We report a pilot on the same 18-report corpus comparing the frame profiles that two foundation-model backends from different families produce for each report. The two backends do not commit to a single shared frame vocabulary, so the comparison reported below is the per-participant alignment between their frame sets—defined for every report.

#### 5.4.2 Protocol

The corpus comprised of 18 verbal reports from the male-embodiment catcalling deployment (one report per participant ranging from a single six-word utterance to a multi-clause narrative). Each report is processed end-to-end through the ISIT pipeline under two foundation-model backends from different model families (Gemini 3.1 Pro and Claude Opus 4.6) with the input specification held fixed: “diagnose verbal reports … use ISIT to create per-participant frame profiles and frame clusters,” H = {IMPLICATURE, IMPACT, COERCION, CAUSALITY, ToM, NEURO, PERSPECTIVE, DIAGNOSIS}, M = {ACTIVE-INFERENCE, ACT-AS, ISIT, ABDUCTIVE-DIAGNOSIS}. The two backends do not commit to the same frame vocabulary, so the cross-backend comparison is computed per participant as the alignment between their two frame sets—the precision of one backend’s frames against the other’s and the recall of the other’s frames, combined as an F1—rather than as a cosine over a fixed common vector; this alignment credits the frames each backend actually asserts and is defined for every one of the 18 reports.

*Criterion (within-versus-between consistency).* The appropriate test for a score-level consistency claim is not whether a within-report cross-backend similarity exceeds a published between-participant convergent-validity correlation—those are quantities on different scales—but whether the same report yields a more cross-backend-similar profile than two different reports do, under a frame alignment that credits semantically adjacent frames rather than requiring identical labels. The present pilot reports that contrast directly: it compares each report’s cross-backend alignment against a baseline of mismatched report pairs (for results, see Section 5.4.3).

#### 5.4.3 Results

The two backends produce frame profiles of different richness over a partly shared vocabulary. Claude returns the richer profile—a mean of 3.2 frames per report, drawing on avoidance, fear, appraisal, anger, embodied, and gender frames—while Gemini returns a sparser one, a mean of 1.2 frames per report concentrated on avoidance, fear, vigilance, and confrontation; the two share avoidance, fear, and confrontation, and Gemini systematically omits the richer tacit frames that Claude adds (anger, embodied, gender, and appraisal). The per-participant alignment reflects this asymmetry: across all 18 reports the precision of Gemini’s frames against Claude’s is high (mean 0.91—almost every frame Gemini asserts is one Claude also asserts), while the recall of Claude’s frames by Gemini is lower (mean 0.58), for a mean F1 of 0.70. To confirm that this agreement is specific to the report rather than a generic effect of the shared vocabulary, we compared it against a between-report baseline—the alignment of each participant’s profile under one backend to every other participant’s profile under the other. That baseline is substantially lower (mean F1 0.48); every one of the 18 reports aligns more closely with its own cross-backend counterpart than with the average mismatched report, and the difference is significant (Mann–Whitney U test, p ≈ 0.002). The same report therefore yields a markedly more cross-backend-similar profile than two different reports do—the comparison appropriate to a backend-stability claim. The disagreement between the backends is therefore concentrated in tacit content, not in error: the sparser backend withholds beyond-stated frames that the richer one asserts, but rarely asserts a frame the other rejects. This is the cross-model face of the precision-versus-recall trade-off that the DEEPJUDGE verification below quantifies directly: the conservative backend buys precision by recovering less implicit content, the generous one recovers more at a small precision cost.

*Verifying the extraction with DEEPJUDGE.* The alignment shows where the two backends agree and where they differ. Still, neither it nor any cross-backend agreement can establish whether the frames a backend places are warranted by the report in the first place—two backends can agree on a frame that is wrong. The frames that carry ISIT’s signal are precisely the ones the report does not state. Warrant is the harder and more consequential question for a tacit extractor. To put it on an auditable footing we extended the analysis with DEEPJUDGE, an experimental method in the POLANYI++ registry that adjudicates every extracted frame against its source report on a four-verdict warrant lattice: explicitly stated; tacitly warranted—admissible only when a named pragmatic mechanism (presupposition, implicature, pragmatic impact, theory-of-mind, or causality) licenses the frame from a specific span of the report; weakly supported; or unwarranted (projection). The lattice is deliberately not a stated-only check—that would score the method’s core capability as error—but the unwarranted verdict still rejects projection, so the criterion credits inference without licensing invention.

DEEPJUDGE runs as a panel rather than a single rubric prompt. On the 18-report corpus a foundation-model judge adjudicated every frame and two human annotators then independently re-adjudicated the entire set—a full rather than sampled human verification, which is feasible at this scale; for larger corpora DEEPJUDGE admits a sampled human pass over a stratified subset of frames. Agreement among the panel was high. Collapsing the four-verdict lattice to the binary that matters for over-projection—whether a frame earned a firm warrant (explicitly stated, or tacitly warranted by a named mechanism) or fell short of one (weakly supported or unwarranted)—the two human annotators agreed with each other on 99% of frames. The model judge agreed with the human annotators on 96%. Separately, the verdicts are checkable rather than taken on trust: because every tacit verdict names the licensing mechanism it rests on, a reviewer can verify that mechanism against the cited span in the report.

Judged on the warrant criterion, frame-level precision is high for both backends—0.96 for the more generous backend (Claude) and 0.95 for the more conservative one (Gemini)—with negligible residual over-projection: at most a single frame (0.02) judged unwarranted even tacitly, and that one confirmed unanimously by both human raters. On a stated-only criterion the same two extractions score only 0.52 and 0.82; the gap between the criteria is the tacit yield, 0.45 versus 0.14, which measures the warranted beyond-stated content the extraction recovers and is the quantity that distinguishes POLANYI++ extraction from surface frame-spotting. The two backends are therefore near-identical in precision but differ sharply in yield: the conservative backend buys precision by recovering less implicit content, the generous one recovers more at a small precision cost—the same trade-off a cautious versus a permissive human reader face.

DEEPJUDGE improves ISIT in three concrete ways. First, it supplies the accuracy warrant ISIT previously lacked: the frames entering a Situation Profile are not merely reproducible but adjudicated, each carrying a named licensing mechanism and a human-verified verdict, so a profile dimension can be traced to the specific inference that justifies it. Second, running as a gate ahead of scoring it removes the small residual of unwarranted frames before they enter a profile, so over-projection cannot distort a score, and it admits weakly-supported frames only provisionally, excluding them from the headline value. Third, it turns the manual quality-assurance loop of Section 3.4 from an informal precaution into a specified procedure with an inter-rater statistic—the form of cross-checking that the SUPERVISED delegation-risk verdict actually demands. In the configuration reported here DEEPJUDGE is applied *post hoc*, as a verification of the case-study extraction; applied inline as a gate, the same method becomes a standing precision control on ISIT scoring.

The connection to DARKSIDE closes the loop. DARKSIDE’s audit (Section 3.1) classifies the commitments a report makes not only for coherence but on a warrant axis—warranted, misattributed, fabricated, or unattested—and folds a fabrication rate into its delegation-risk verdict. An unwarranted DEEPJUDGE frame is, on that axis, a fabricated commitment, so DEEPJUDGE’s over-projection rate is exactly the extraction-level fabrication signal DARKSIDE consumes; the near-zero rate measured here is why these reports receive the SUPERVISED verdict rather than UNSAFE. Verdict and method are thus two ends of one mechanism: DARKSIDE certifies that automated scoring is admissible only under human cross-check, and DEEPJUDGE is the auditable form that cross-check now takes. The full verification sheet—per-frame verdict, licensing mechanism, and both human passes—is provided in the Supplementary materials (Frame-level extraction verification; Supplementary Tables S3, S4).

## 6 Conclusion and future work

The Implicit Situation Intensity Test is a frame-clustering implicit-measure method built on the POLANYI++ extraction backbone, designed to fill the verbal-channel gap in the cognitive-science measurement ecosystem. It produces per-participant Situation Profiles—typed numerical vectors over a Frame Ontology, with each frame’s salience scored on a normalized scale—designed for direct integration with explicit measures from standardized questionnaires, implicit measures from physiological sensors, and participant-profile covariates. ISIT is not an automated grounded theory: it does not perform open or axial coding, it does not produce corpus-level themes, and it does not stand alone as a qualitative analysis. It is one measurement channel in a multivariate cognitive-science design, and its value depends on how it integrates with the other channels.

The contribution of this study includes ISIT’s explicit relationship to the POLANYI++ extraction backbone; ISIT’s operational distinction from grounded theory and its computational variants; and ISIT’s integration recipe with other measurement channels. The empirical anchor demonstrates the method on cognitive-science problems where the verbal channel matters and integration with other channels is essential. The study that remains—convergent, divergent, and predictive validity studies; multi-modal integration validation—is concrete and is set out as a defined roadmap. Beyond demonstrating the method, the article verifies the extraction itself: a tacit-aware adjudication (DEEPJUDGE), human-checked, shows that the frames entering Situation Profiles are warranted rather than merely reproducible. Frame-level precision is high and over-projection near zero—the accuracy footing ISIT previously lacked—and the same report yields markedly more similar profiles across backends than different reports do.

An application and validation roadmap includes the following:

- A standard psychometric validation program: a battery comprising at least one IAT relevant to the measurement context, WEAT measurements over the relevant text corpus where applicable, validated explicit self-report scales for the constructs in question, and ISIT Mode A scoring of verbal reports from the same participants, run on a single sample of n≥100 participants. Convergent and divergent correlations are then computed at the frame level, and incremental-validity regressions test whether ISIT provides measurement information beyond what the questionnaires already provide.
- ISIT scores from a controlled-context task (a VR study, a clinical interview, and an HCI usability test) should predict subsequent task-relevant behavior better than, or incrementally to, existing explicit-measure baselines. This is the strongest test of the construct claim and the highest-effort item on the roadmap.
- A purpose-designed study collecting verbal reports, standardized questionnaire data, and at least one channel of physiological measurement (GSR or eye-tracking being the lowest-cost candidates) on the same participants, then running the full multivariate pipeline of Section 5 to test (i) that the channel-level measurements are mutually informative, (ii) that ISIT’s contribution to the integrated model is incremental, and (iii) that the integrated model predicts behavioral outcomes more accurately than any single channel.
- A comparative study with LLMs in zero-shot or few-shot direct scoring of frames from verbal reports—the emerging “LLM-as-judge” protocol—which is the most parsimonious automation of the verbal-report-to-score pipeline. ISIT differs from this protocol in three architecturally significant ways: (i) ISIT operates on the structured POLANYI++ XKG, not on raw text, so LLM scoring is conditioned on a formal extraction; (ii) ISIT’s Frame Ontology is induced (Mode B) or supplied (Mode A) explicitly, not constructed implicitly by the LLM at scoring time; (iii) ISIT’s scoring algorithm is transparent and decomposable into the three weighted components (manifestation, affective alignment, and contextual influence), each of which can be inspected and debugged. The LLM-as-judge protocol is faster and conceptually simpler, but its outputs are more opaque and its variance under prompt drift is higher. Comparing ISIT to LLM-as-judge on a fixed corpus, with explicit measurements of inter-run variance under prompt perturbation, is the relevant future study.

## Data Availability

Publicly available datasets were analysed in this study. This data can be found at: https://github.com/aldogangemi/catcalling.

## References

[ref1] Bagby R. M. Parker J. D. A. Taylor G. J. (1994). The twenty-item Toronto alexithymia scale–I. Item selection and cross-validation of the factor structure. J. Psychosom. Res. 38, 23–32. 10.1016/0022-3999(94)90005-1 8126686

[ref3] Barrett L. F. (2017). The theory of constructed emotion: an active inference account of interoception and categorization. Soc. Cogn. Affect. Neurosci. 12, 1–23. doi: 10.1093/scan/nsw154, 27798257 PMC5390700

[ref4] Barsalou L. W. (2009). Simulation, situated conceptualization, and prediction. Philos. Trans. R. Soc. B 364, 1281–1289. doi: 10.1098/rstb.2008.0319, 19528009 PMC2666716

[ref9001] Barsalou L. W. (2020). Challenges and opportunities for grounding cognition. Journal of Cognition, 3:31. doi: 10.5334/joc.116, 33043241 PMC7528688

[ref5] Birks M. Mills J. (2015). Grounded Theory: A Practical guide. 2nd Edn. Thousand Oaks, in California (Stati Uniti): SAGE Publications.

[ref6] Caliskan A. Bryson J. J. Narayanan A. (2017). Semantics derived automatically from language corpora contain human-like biases. Science 356, 183–186. doi: 10.1126/science.aal4230, 28408601

[ref7] Carlsen H. B. Ralund S. (2022). Computational grounded theory revisited: from computer-led to computer-assisted text analysis. Big Data Soc. 9, 1–14. doi: 10.1177/20539517221080146

[ref8] Charmaz K. (2014). Constructing Grounded Theory. 2nd Edn. United States: SAGE Publications.

[ref19] Dai S. C. Xiong A. Ku L. W. (2023). LLM-in-the-loop: Leveraging large language model for thematic analysis. In Findings of the association for computational linguistics: EMNLP 2023 (pp. 9993–10001). doi: 10.18653/v1/2023.findings-emnlp.669

[ref9] Damasio A. R. (1994). Descartes’ error: Emotion, Reason, and the human brain. Putnam.

[ref10] De Giorgis S. Gangemi A. Russo A. (2025). Neurosymbolic graph enrichment for grounded world models. Inf. Process. Manag. 62, 104–127. doi: 10.1016/j.ipm.2025.104127

[ref11] Ekman P. (1992). An argument for basic emotions. Cognit. Emot. 6, 169–200.

[ref12] Ericsson K. A. Simon H. A. (1984). Protocol Analysis: Verbal Reports as data. Cambridge, MA: MIT Press.

[ref13] Fillmore C. J. (2006). “Frame semantics,” in Cognitive Linguistics: Basic Readings, ed. Geeraerts D. (Berlin: De Gruyter Mouton), 373–400.

[ref14] Fox M. C. Ericsson K. A. Best R. (2011). Do procedures for verbal reporting of thinking have to be reactive? A meta-analysis and recommendations for best reporting methods. Psychol. Bull. 137, 316–344. doi: 10.1037/a0021663 21090887

[ref15] Frijda N. H. (1986). The Emotions. Cambridge University Press.

[ref9101] Gangemi A. Bottazzi E. (2026). Walking on the DARKSIDE. arXiv. preprint arXiv:2608.23370.

[ref16] Glaser B. G. Strauss A. L. (2009). The discovery of grounded theory: Strategies for qualitative research. Piscataway, New Jersey: Transaction publishers.

[ref17] Graham J. Haidt J. Koleva S. Motyl M. Iyer R. Wojcik S. P. . (2013). Moral foundations theory: the pragmatic validity of moral pluralism. Adv. Exp. Soc. Psychol. 47, 55–130. doi: 10.1016/B978-0-12-407236-7.00002-4

[ref18] Greenwald A. G. McGhee D. E. Schwartz J. L. K. (1998). Measuring individual differences in implicit cognition: the implicit association test. J. Pers. Soc. Psychol. 74, 1464–1480. 10.1037//0022-3514.74.6.1464 9654756

[ref2] He K. Meng Q. Feng X. Zhang X. Nissim M. (2026). LLM-enhanced computational grounded theory and the triadic dynamics of human-AI-society interaction. Theory and Society, 55:66. doi: 10.1007/s11186-026-09734-7

[ref20] Lazarus R. S. (1991). Emotion and Adaptation. Oxford University Press.

[ref21] Lindquist K. A. Wager T. D. Kober H. Bliss-Moreau E. Barrett L. F. (2012). The brain basis of emotion: a meta-analytic review. Behav. Brain Sci. 35, 121–143. doi: 10.1017/S0140525X11000446, 22617651 PMC4329228

[ref22] Lucifora C. Gangemi A. Miglietta C. Schembri M. Vicario C. M. Miglietta C. P. . (2025). Virtual embodiment increases male sensitivity to catcalling experiences. Sci. Rep. 15:35376. doi: 10.1038/s41598-025-19418-4, 41068335 PMC12511580

[ref23] Nelson L. K. (2020). Computational grounded theory: a methodological framework. Sociol. Methods Res. 49, 3–42. doi: 10.1177/0049124117729703

[ref24] Nisbett R. E. Wilson T. D. (1977). Telling more than we can know: verbal reports on mental processes. Psychol. Rev. 84, 231–259.

[ref25] Ortony A. Clore G. L. Collins A. (1988). The Cognitive Structure of Emotions. Cambridge University Press.

[ref26] Panksepp J. (1998). Affective Neuroscience: The Foundations of human and animal Emotions. Oxford University Press.

[ref27] Reiter R. (1981). “On closed world data bases,” in Readings in artificial Intelligence, eds. Webber B. L. Nilsson N. J. (Palo Alto, California United States: Morgan Kaufmann), 119–140.

[ref28] Russell J. A. (2003). Core affect and the psychological construction of emotion. Psychol. Rev. 110, 145–172. doi: 10.1037/0033-295X.110.1.145, 12529060

[ref29] Schwartz S. H. (1992). Universals in the content and structure of values: theoretical advances and empirical tests in 20 countries. Adv. Exp. Soc. Psychol. 25, 1–65. doi: 10.1016/s0065-2601(08)60281-6

[ref30] Schwitzgebel E. (2008). The unreliability of naive introspection. Philos. Rev. 117, 245–273. doi: 10.1215/00318108-2007-037

[ref31] Schwitzgebel E. (2011). Perplexities of Consciousness. Cambridge: MIT Press.

[ref32] Spielberger C. D. Gonzalez-Reigosa F. Martinez-Urrutia A. Natalicio L. F. Natalicio D. S. (1971). The state-trait anxiety inventory. Revista Interamericana de Psicologia/Interamerican J. Psycho. 5, 3–4.

[ref33] Strauss A. Corbin J. (1990). Basics of Qualitative Research: Grounded Theory Procedures and Techniques. Thousand Oaks, in California (Stati Uniti): SAGE Publications.

