# Supplementary material for: ISIT: a frame-clustering implicit measure of situated experience
Source: Front Psychol. 2026 Oct 5;17:1883850. doi: 10.3389/fpsyg.2026.1883850 (PMC13679706; doi:10.3389/fpsyg.2026.1883850)
Supplement: Supplementary file 1 [file Supplementary_file_1.DOCX]

**Supplementary Materials**

Figure 1

**Figure 1 caption.** *The POLANYI++ extraction pipeline as the function* $E=f(I,O,S,B,H,T,M,U)$*.* A schematic of the four modes (EXTRACT, DISCOVER, REVISE, ALIGN) with the inputs ($I$ modality, $O$ inferential operator, $S$ source, $T$ task, $U$ ontology prior) feeding into Mode 1 (EXTRACT), which applies the heuristic set $H$ to produce the base graph $B$ and then augments it into the extended graph $E$ via heuristic-annotated triples. The separation between $B$ and $E$ is shown as a hard boundary; every inferred triple in $E$ carries a provenance arrow back to the heuristic that produced it and ultimately to the verbatim text in $S$. Suggested format: layered horizontal layout with $S$ on the left, the four modes vertically stacked in the middle, and the extended knowledge graph $E$ on the right, with the audit-chain arrows shown in a distinct colour.

Figure 2

**Figure 2 caption.** *ISIT Mode A workflow: from a single participant’s extended knowledge graph to a Situation Profile.* Phase 1 (Frame extraction) collects frames from heuristic-annotated triples in $E$. Phase 2 (Frame clustering) maps the raw frames against a Frame Ontology — either an externally supplied anchor structure or the ontology induced by Mode B Phase 0 — assigning each frame to a nearest cluster and admitting emergent frames as extensions. Phase 3 (ISIT scoring) computes the per-frame composite score from three evidence components ($S_{M}$, $S_{E}$, $S_{I}$). Phase 4 (Profile assembly) packages the scored frames, the per-frame perspectival qualia inherited from QUALECOMPOSITION, and the DARKSIDE audit summary into a typed isit:SituationProfile. Suggested format: left-to-right pipeline with the four phase boxes; below each box, a small example of the artefact produced (a list of raw frames, a small Frame Ontology fragment, a vector of frame scores, the final Situation Profile object).

Figure 3

**Figure 3.** *ISIT frame scoring procedure (CalculateISITFrameScore).* Inputs: the target frame, the verbal report text, the set of identified emotions, the vector of contextual data, the extended knowledge graph produced by POLANYI++, and the theoretical score bounds. Steps 1–3 compute the three substrate-evidence components (Direct Manifestation, Affective Alignment, Contextual Influence), apply optional frame-specific modifiers, and combine them as a weighted sum with default weights $(w_{M},w_{E},w_{I})=(0.40,0.30,0.30)$. Step 4 applies a contextual adjustment using the CUECOHERENCE cross-channel verdict and the DARKSIDE cross-temporal audit. Step 5 normalises to $[-1,+1]$ by min–max scaling against the theoretical bounds.

Figure 4

**Figure 4 caption.** *ISIT Mode B: induction of the shared Frame Ontology from the union of participants’ extended knowledge graphs.* Top: $n$ participants each produce a verbal report $S_{i}$, processed by POLANYI++ into an extended knowledge graph $E_{i}$. Middle: Phase 0 of Mode B unions the per-participant frame sets into the shared Frame Ontology $\mathrm{FO}=\bigcup_{i} \mathrm{frames}(E_{i})$, distinguishing *anchor frames* (present in two or more groups, shown in solid colour) from *emergent frames* (group-specific, shown in hatched colour). Bottom: each $E_{i}$ is scored against the induced $\mathrm{FO}$ to produce a sparse Situation Profile $\mathrm{SP}_{i}$; the $\mathrm{SP}_{i}$ vectors stack into a per-group profile matrix used for statistical comparison (inter-rater agreement, group contrasts, mixed-model analyses). Suggested format: three vertically stacked rows with arrows between them; the anchor/emergent distinction is colour-coded in the middle row.

Figure 5

**Figure 5 caption.** *ISIT as the verbal-channel implicit measure in a four-channel cognitive-science design.* Schematic of the integrated multivariate analysis: four channels feed into a single per-participant measurement vector — the *verbal channel* (verbal report → POLANYI++ extraction → ISIT Situation Profile); the *explicit channel* (standardised questionnaires producing per-participant scores); the *physiological/behavioural channel* (GSR, HRV, eye-tracking, EEG/fMRI producing time-aligned features or summary statistics); and the *participant-profile channel* (demographic and clinical covariates). The four-channel vector is the input to the analytical model (linear mixed model, structural equation model, or multi-modal fusion architecture depending on study design). Suggested format: four horizontal channel lanes converging into a single merging block labelled “multivariate model”, with the dependent variable (typically a behavioural outcome) at the right.

Figure 6

**Figure 6 caption.** *Empirical anchoring of ISIT in the male-embodiment catcalling study (Lucifora et al., 2025).* Top: extraction backbone schematic, showing the seven heuristics applied (BASE, PRESUPPOSITION, IMPLICATURE, IMPACT, COERCION, CAUSALITY, ToM) and the resulting per-participant extended knowledge graph. Middle: the 37-indicator Frame Ontology organised in four domains (13 emotional-state, 5 behavioural-response, 15 violence-perception, 4 semantic-marker). Bottom: integration with explicit measures (embodiment questionnaire, Ekman emotion-intensity ratings) showing convergent correlations of immersion frames with embodiment subscales and differential correlation patterns of safety-related frames with anger/sadness vs fear. Suggested format: a three-row layout with arrows from extraction to FO to integrated multivariate model.

Table 1

**Table 1.** POLANYI++ heuristics applied in verbal-report analysis.

| Heuristic | Function | Frame contribution |
| --- | --- | --- |
| BASE | Base extraction | Text-grounded entities and events; the empirical foundation for all subsequent inference |
| PRESUPPOSITION | Background-assumption detection | Frames presupposed but not stated (e.g., “he started again” presupposes prior occurrence) |
| IMPLICATURE | Pragmatic-inference extraction | Gricean-implicated frames, including scalar and ironic implicatures |
| IMPACT | Multi-layer impact analysis | Emotional, social, cognitive, and bodily-impact frames; the principal source of emotion frames |
| COERCION | Semantic coercion | Metaphorical, metonymical, and symbolic-meaning frames |
| CAUSALITY | Causal reasoning | Causal-attribution frames (e.g., victim-blame, guilt induction) |
| ToM | Theory of mind | Motivational, intentional, and epistemic frames for participant, victim, and other agents |
| PERSPECTIVE | Perspectival framing | Reference-frame and perspective-shift frames; reliability levels of narrator |
| HYPOTHETICAL | Counterfactual and hypothetical marking | Frames marked as conditional or counterfactual |
| MODALITY | Modal logic | Strength and certainty modulation of commitments |
| TACITTHEORYKNOWLEDGE | Tacit theoretical commitments | Background theoretical assumptions evident in the participant’s framing |
| COGBIAS | Cognitive-bias detection | Recognition of standard biases in the report’s structure |
| VISUALSEMIOTICS | (Multimodal) | Compositional and semiotic frames in image-accompanied reports |

Table 2

**Table 2.** ISIT scoring components, default weights, theoretical justification, and generalisable data sources.

| Component | Default weight | Theoretical justification | Generalisable data sources |
| --- | --- | --- | --- |
| Direct Frame Manifestation | ≈ 40% | Frame semantics (Fillmore, 2006) | Verbal/written reports (linguistic cues) |
| Affective Alignment | ≈ 30% | Somatic marker hypothesis (Damasio, 1994); appraisal theory (Lazarus, 1991) | Verbal/written reports (emotional lexicon, QUALECOMPOSITION projections) |
| Contextual Influence | ≈ 30% | Situated/embodied cognition (Barsalou, 2009) | Embodiment questionnaires (VR), physiological sensors (GSR, HRV), eye-tracking, keystroke logs, fMRI/EEG; CUECOHERENCE/DARKSIDE audit verdicts |

# Frame-level extraction verification (DEEPJUDGE method)

Every frame extracted by POLANYI++ from the 18 catcalling reports was adjudicated against its source report by the experimental DEEPJUDGE method on a four-verdict warrant lattice: Explicit (stated in the report), Tacit (not stated but licensed by a named pragmatic mechanism — presupposition, implicature, pragmatic impact, theory-of-mind, or causality), Weak (a strained or under-determined tacit reading), or Unwarranted (projection). A foundation-model judge produced the verdicts and licensing mechanisms; two human annotators then independently re-adjudicated all 78 frames. Table 3 summarises the precision profile per backend; Table 4 gives the full per-frame sheet with the judge verdict, the licensing mechanism, and both human passes. Precision on the tacit criterion counts Explicit and Tacit verdicts as correct; the tacit yield is the proportion of frames warranted tacitly (i.e. recovered beyond what the report states); over-projection is the proportion judged Unwarranted.

**Table 3. DEEPJUDGE frame-level verdict distribution and warrant-criterion precision by backend, human-verified consensus.**

| **Backend** | **Frames (n)** | **Explicit (n / %)** | **Tacit (n / %)** | **Weak (n / %)** | **Unwarranted (n / %)** | **Warrant-criterion precision** | **Tacit yield** | **Over-projection** |
| --- | --- | --- | --- | --- | --- | --- | --- | --- |
| **Claude** | **56** | **29 / 52%** | **25 / 45%** | **1 / 2%** | **1 / 2%** | **0.96** | **0.45** | **0.02** |
| **Gemini** | **22** | **18 / 82%** | **3 / 14%** | **0–1 / 0–4%** | **0–1 / 0–4%** | **0.95–1.00** | **0.14** | **0.00** |

***Note.* Warrant-criterion precision = (Explicit + Tacit) / Frames(n). The columns labelled “Explicit (n / %)” and “Tacit (n / %)” describe the *composition* of warranted frames — what proportion of each backend’s output is explicitly stated versus inferred — not its accuracy. Both backends are near-perfect on the precision criterion (0.95–0.96); they differ sharply in tacit yield (0.45 vs 0.14), which measures how much beyond-stated warranted content each extraction recovered. Both have near-zero over-projection (≤ 0.02). The 0–4% range for Gemini’s Weak and Unwarranted entries reflects a single contested verdict between the two human raters (participant 8, Gemini, *Confrontation*: one rater Tacit, one Weak; the judge had marked it Unwarranted), which is the source of the 0.95–1.00 range on the precision column.**

**Inter-annotator agreement (the two human passes) on the warranted-versus- not distinction was 77/78 = 98.7%; judge–human agreement was 75/78 = 96%.**

**Table 4.** Full DEEPJUDGE verification sheet: per-frame judge verdict, licensing mechanism, and both human passes (78 frames).

| **Pt.** | **Backend** | **Frame** | **Judge verdict** | **Licensing mechanism** | **Human 1** | **Human 2** |
| --- | --- | --- | --- | --- | --- | --- |
| 1 | Claude | avoidance | Explicit | stated | Explicit | Explicit |
| 1 | Claude | gender_conflict | Explicit | stated | Explicit | Explicit |
| 1 | Claude | appraisal | Explicit | stated | Explicit | Explicit |
| 1 | Claude | fear | Tacit | ToM/IMPACT | Tacit | Tacit |
| 1 | Gemini | Avoidance | Explicit | stated | Explicit | Explicit |
| 2 | Claude | fear | Explicit | stated | Explicit | Explicit |
| 2 | Claude | avoidance | Explicit | stated | Explicit | Explicit |
| 2 | Claude | appraisal | Explicit | stated | Explicit | Explicit |
| 2 | Claude | embodied | Explicit | stated | Explicit | Explicit |
| 2 | Gemini | Avoidance | Explicit | stated | Explicit | Explicit |
| 2 | Gemini | Fear | Explicit | stated | Explicit | Explicit |
| 3 | Claude | avoidance | Explicit | stated | Explicit | Explicit |
| 3 | Claude | appraisal | Tacit | IMPLICATURE | Tacit | Tacit |
| 3 | Claude | embodied | Weak | IMPACT? | Tacit | Tacit |
| 3 | Claude | fear | Tacit | ToM | Tacit | Tacit |
| 3 | Gemini | Vigilance | Explicit | stated | Explicit | Explicit |
| 4 | Claude | appraisal | Explicit | stated | Explicit | Explicit |
| 4 | Claude | avoidance | Explicit | stated | Explicit | Explicit |
| 4 | Claude | embodied | Explicit | stated | Explicit | Explicit |
| 4 | Claude | fear | Tacit | IMPACT | Tacit | Tacit |
| 4 | Gemini | Vigilance | Tacit | IMPLICATURE | Tacit | Tacit |
| 5 | Claude | appraisal | Tacit | IMPLICATURE | Tacit | Tacit |
| 5 | Claude | fear | Tacit | ToM | Tacit | Tacit |
| 5 | Claude | avoidance | Weak | - | Tacit | Tacit |
| 5 | Gemini | Vigilance | Explicit | stated | Explicit | Explicit |
| 6 | Claude | avoidance | Explicit | stated | Explicit | Explicit |
| 6 | Claude | fear | Explicit | stated | Explicit | Explicit |
| 6 | Claude | appraisal | Explicit | stated | Explicit | Explicit |
| 6 | Gemini | Avoidance | Explicit | stated | Explicit | Explicit |
| 6 | Gemini | Fear | Explicit | stated | Explicit | Explicit |
| 7 | Claude | appraisal | Tacit | PRESUPPOSITION | Tacit | Tacit |
| 7 | Claude | fear | Tacit | IMPACT | Tacit | Tacit |
| 7 | Claude | embodied | Tacit | IMPACT | Tacit | Tacit |
| 7 | Claude | avoidance | Tacit | IMPLICATURE | Tacit | Tacit |
| 7 | Claude | anger | Unwarranted | - | Unwarranted | Unwarranted |
| 7 | Gemini | Fear | Tacit | IMPACT | Tacit | Tacit |
| 8 | Claude | anger | Explicit | stated | Explicit | Explicit |
| 8 | Claude | embodied | Tacit | IMPACT | Tacit | Tacit |
| 8 | Gemini | Confrontation | Unwarranted | - | Tacit | Weak |
| 9 | Claude | avoidance | Explicit | stated | Explicit | Explicit |
| 9 | Claude | appraisal | Tacit | CAUSALITY | Tacit | Tacit |
| 9 | Claude | fear | Tacit | ToM | Tacit | Tacit |
| 9 | Gemini | Avoidance | Explicit | stated | Explicit | Explicit |
| 10 | Claude | avoidance | Explicit | stated | Explicit | Explicit |
| 10 | Claude | fear | Tacit | IMPACT | Tacit | Tacit |
| 10 | Gemini | Avoidance | Explicit | stated | Explicit | Explicit |
| 11 | Claude | avoidance | Explicit | stated | Explicit | Explicit |
| 11 | Claude | appraisal | Tacit | CAUSALITY | Tacit | Tacit |
| 11 | Claude | embodied | Tacit | IMPACT | Tacit | Tacit |
| 11 | Claude | fear | Tacit | ToM | Tacit | Tacit |
| 11 | Claude | anger | Tacit | IMPLICATURE | Tacit | Tacit |
| 11 | Gemini | Avoidance | Explicit | stated | Explicit | Explicit |
| 11 | Gemini | Fear | Tacit | IMPACT | Tacit | Tacit |
| 12 | Claude | avoidance | Explicit | stated | Explicit | Explicit |
| 12 | Claude | fear | Explicit | stated | Explicit | Explicit |
| 12 | Gemini | Avoidance | Explicit | stated | Explicit | Explicit |
| 12 | Gemini | Fear | Explicit | stated | Explicit | Explicit |
| 13 | Claude | avoidance | Explicit | stated | Explicit | Explicit |
| 13 | Claude | appraisal | Explicit | stated | Explicit | Explicit |
| 13 | Claude | embodied | Weak | - | Weak | Weak |
| 13 | Claude | fear | Tacit | ToM | Tacit | Tacit |
| 13 | Gemini | Avoidance | Explicit | stated | Explicit | Explicit |
| 14 | Claude | avoidance | Explicit | stated | Explicit | Explicit |
| 14 | Claude | appraisal | Explicit | stated | Explicit | Explicit |
| 14 | Claude | fear | Tacit | ToM/IMPACT | Tacit | Tacit |
| 14 | Gemini | Avoidance | Explicit | stated | Explicit | Explicit |
| 15 | Claude | avoidance | Explicit | stated | Explicit | Explicit |
| 15 | Claude | appraisal | Explicit | stated | Explicit | Explicit |
| 15 | Claude | fear | Tacit | PRESUPPOSITION | Tacit | Tacit |
| 15 | Gemini | Avoidance | Explicit | stated | Explicit | Explicit |
| 16 | Claude | avoidance | Explicit | stated | Explicit | Explicit |
| 16 | Claude | appraisal | Tacit | IMPLICATURE | Tacit | Tacit |
| 16 | Claude | fear | Tacit | PRESUPPOSITION | Tacit | Tacit |
| 16 | Gemini | Avoidance | Explicit | stated | Explicit | Explicit |
| 17 | Gemini | Unaffected | Explicit | stated | Explicit | Explicit |
| 18 | Claude | anger | Explicit | stated | Explicit | Explicit |
| 18 | Claude | confrontation | Explicit | stated | Explicit | Explicit |
| 18 | Gemini | Confrontation | Explicit | stated | Explicit | Explicit |
